# Risk Stratification and Treatment in Smoldering Multiple Myeloma

**DOI:** 10.3390/cells11010130

**Published:** 2021-12-31

**Authors:** Tyler Lussier, Natalie Schoebe, Sabine Mai

**Affiliations:** 1 Department of Physiology and Pathophysiology, University of Manitoba, Cancer Care Manitoba Research Institute, Winnipeg, MB R3E 0V9, Canada; lussiert@myumanitoba.ca (T.L.); ns864@cam.ac.uk (N.S.); 2 Faculté des Sciences, Université de Saint-Boniface, Winnipeg, MB R2H 0H7, Canada; 3 Faculty of Biosciences, University of Heidelberg, 69117 Heidelberg, Germany

**Keywords:** smoldering multiple myeloma, risk stratification, treatment

## Abstract

Smoldering multiple myeloma is a heterogeneous asymptomatic precursor to multiple myeloma. Since its identification in 1980, risk stratification models have been developed using two main stratification methods: clinical measurement-based and genetics-based. Clinical measurement models can be subdivided in three types: baseline measurements (performed at diagnosis), evolving measurements (performed over time during follow-up appointments), and imaging (for example, magnetic resonance imaging). Genetic approaches include gene expression profiling, DNA/RNA sequencing, and cytogenetics. It is important to accurately distinguish patients with indolent disease from those with aggressive disease, as clinical trials have shown that patients designated as “high-risk of progression” have improved outcomes when treated early. The risk stratification models, and clinical trials are discussed in this review.

## 1. Introduction

Smoldering multiple myeloma (SMM) or asymptomatic multiple myeloma (AMM) is a heterogeneous asymptomatic precursor to multiple myeloma (MM) [1]. It is the intermediate stage between monoclonal gammopathy of undetermined significance (MGUS) and MM, where there is a subset of patients with indolent disease and a subset with a progressive disease [1,2,3]. SMM has an overall higher risk of progression to MM compared to MGUS [2], at 10% per year versus 1% per year.

The term SMM was first proposed in 1980 by Kyle and Greipp after reviewing all of the MM patients at the Mayo Clinic before 1 January 1974 [4]. This led to the discovery of six patients who met the diagnosis criteria for MM at the time of ≥10% abnormal bone marrow plasma cells (BMPCs) and a serum M-protein concentration >3 g/dL [4], but they did not present with the characteristic CRAB (hypercalcemia, renal failure, anemia, and lytic bone lesions) features of MM [1,4]. Therefore, these patients were said to have SMM as an analogy to smoldering acute leukemia [4], an asymptomatic precursor to acute leukemia.

In 2003, the International Myeloma Working Group (IMWG) proposed new criteria for the diagnosis of MM as well as its related precursors of MGUS and SMM [5]. SMM was defined here as M-protein ≥ 3 g/dL and/or clonal BMPCs ≥ 10% with no related end-organ damage [5], which are the CRAB features, or recurrent bacterial infections related to the malignancy. The diagnostic criteria for SMM as well as MM and MGUS were updated by the IMWG in 2014 [1]. Diagnosis of SMM now requires the absence of myeloma-defining events [1], specifically the CRAB features and nonrecurrent bacterial infections, as well as serum M-protein (IgG or IgA) ≥ 3 g/dL or urinary M-protein ≥ 500 mg/24 h and/or 10–60% clonal BMPCs. In this new model, SMM patients with an 80% risk of progression at 2 years are now considered to have active MM and should be offered treatment [1]. An 80% risk of progression is found in patients with one or more of the following biomarkers: clonal BMPC ≥ 60%, involved: uninvolved (i:u) serum free light chain ratio (FLCr) ≥ 100, and/or >1 focal lesion on magnetic resonance imaging (MRI). This change was made due to improved treatment options having been developed that are less toxic, as well as the strong signs of improvement with early treatment of asymptomatic high-risk patients [1,6]. This also better aligns this malignancy with others because signs of severe end organ damage such as lytic bone lesions and renal failure are no longer required prior to the commencement of treatment [1]. Properly distinguishing patients who have indolent disease versus those who will progress quickly using risk stratification models is a very important issue as it determines who will be offered treatment and when [1,6].

The Mayo Clinic published a risk stratification model in 2018 using the IMWG 2014 diagnostic criteria [7], which was validated by the IMWG in 2020 [8], called the 2/20/20 model as it uses M-protein > 2 g/dL, BMPC >  20%, and i:u FLCr >  20 as cut-offs. High-risk cytogenetic anomalies (t(4;14) and/or del(17p) and/or hyperdiploidy/+1q or t(14;16)) were found to be a fourth risk factor that improved the model [8]; however, not all patients studied had fluorescence in situ hybridization (FISH) data available.

**Figure 1:**
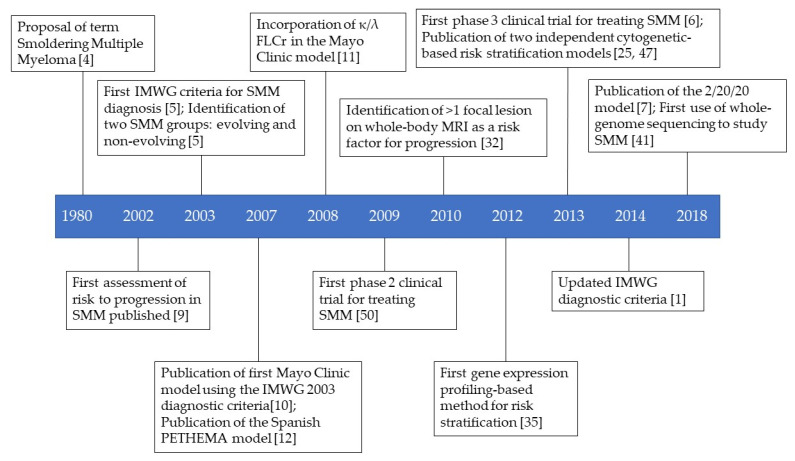
Timeline of key events in the history of SMM risk stratification and treatment.

In this review, the many different models and approaches that are or have been proposed or used to stratify the risk of progression of SMM patients are discussed. Clinical trials of SMM patients are also discussed. Figure 1 provides a timeline of the important events in the assessment of the risk of progression and treatment of SMM.

## 2. Risk Assessment Models

### 2.1. Clinical Markers

#### 2.1.1. Baseline Clinical Measurements

A study from 2002 included 127 SMM patients who were defined with the following parameters [9]: IgG monoclonal component (MC) of 3.6–6.9 g/dL or IgA MC of 2.1–4.9 g/dL, and/or Bence-Jones (BJ) proteinuria > 1 g/24 h, and/or 11–19% BMPCs, and absence of the CRAB features and found that there was a correlation between progression with >10% BMPCs, detectable BJ proteinuria, and IgA isotype.

Kyle’s 2007 retrospective study of patients at the Mayo Clinic between 1970 and 1995 found 276 patients that fulfilled the IMWG 2003 criteria for SMM [10]. These patients were then separated into three groups based on their BMPC% and serum M-protein concentration at diagnosis [10]: group one, BMPC ≥ 10% and M-protein ≥ 3 g/dL; group two, BMPC ≥ 10% and M-protein < 3 g/dL, and group three, BMPC < 10% and M-protein ≥ 3 g/dL. The cumulative risk of progression at 15 years was 87, 70, and 39%, respectively, for groups one, two, and three [10]. The median TTP was respectively two years, eight years, and 19 years for groups one, two, and three [10]. Other significant baseline risk factors from the study for the progression from SMM to MM or amyloidosis include the presence of IgA or of urinary light chain, a reduction in the amount of uninvolved Ig, and the pattern of PC involvement in the BM [10]. Kyle et al. (2007) also found that the overall risk of progression for the first five years is 10% per year, 3% per year for the following five years and 1% per year afterwards [10], where no such change in risk occurs in MGUS. It was later found that baseline *κ*/*λ* FLCr < 1:8 (0.125) or >8 was a significant and independent risk factor [11]. Dispenzieri et al. added this information to Kyle ’s 2007 model using 273/276 patients from the same cohort [10,11]; risk groups one and two could be split into two separate groups with the split group three having similar results, group one with FLCr 0.125–8: 58.8% absolute risk of progression at 10 years, group one with FLCr < 0.125 or >8: 83.8%, group two with FLCr 0.125–8: 58.3%, group two with FLCr < 0.125 or >8: 68.5%, group three with FLCr 0.125–8: 32.2%, group three with FLCr < 0.125 or >8: 33.3%. Dispenzieri et al. then constructed a risk stratification model and found that the cumulative 10-year probability of progression and median TTP for patients with one risk factor was 50% and 10 years, 65% and 5.1 years for those with two risk factors, and 84% and 1.9 years for those with three [11].

A separate study by the Spanish PETHEMA (Programa de Estudio y Tratamiento de las Hemopatías Malignas) group with 93 SMM patients diagnosed using the IMWG 2003 criteria and/or >10 g/L BJ proteinuria measured the amount of immunophenotypically aberrant PCs in the BM (aPC/BMPC) using multiparameter flow cytometry (FC) [12]. Patients with aPC/BMPC ≥ 95% had a significantly shorter TTP compared to those with <95% aPC/BMPC in patients with low MC (<30 g/L) or without immunoparesis [12], which are two risk factors if present. A model was developed for the risk of progression from SMM using the only two independent prognostic risk factors for progression-free survival (PFS) in this study [12], ≥95% aPC/BMPC and immunoparesis. The five-year PFS and median TTP were, respectively, 4% and not reached, 46% and 73 months, and 72% and 23 months for patients with zero, one, and two risk factors [12].

Cherry et al. compared the Dispenzieri’s Mayo Clinic model and Pérez-Persona’s Spanish PETHEMA group model by determining how many of their 77 well-defined SMM patients fit into each risk group in each model [11,12,13]. For their cohort, 38, 35, and four patients were respectively classified as low, intermediate, and high-risk using the Mayo Clinic model, while the Spanish PETHEMA model determined there to be 17, 22, and 38 patients as low, intermediate, and high-risk, respectively [13]. They found that there was significant discordance in classifying patients as high-risk versus non-high-risk (low plus intermediate-risk) as well as low-risk versus non-low-risk (intermediate plus high-risk) [13], there was only a 28.6% (22/77) concordance between both models. This showed that there was, and still is, a need for better stratifying patients with an improved risk model [13].

The Mayo Clinic improved their model through another retrospective study of 586 patients from 1970–2010 diagnosed using the IMWG 2003 criteria [14]. They measured the i:u FLCr and found an optimal cut-point of ≥100 (rounded up from >91 for clinical simplification) [14], which corresponds to ≥100 or ≤0.01 for the *κ*/*λ* FLCr. This gave a high-risk group (≥100) with a 72% risk of progression to MM at two years and a TTP of 15 months compared to 28% two-year risk and a 55-month TTP in the low-risk group (<100) [14].

The quantification of circulating PCs (cPCs) using an immunofluorescent assay performed on fixed peripheral blood mononucleated cells of 91 patients diagnosed with SMM between January 1994 through January 2007 using the IMWG 2003 criteria found that patients with absolute peripheral blood PCs > 5 × 10^6^/L and/or >5% PCs/100 cytoplasmic Ig-positive peripheral blood mononuclear cells had an increased two-year risk of progression (71 versus 24%), decreased median TTP (12 versus 57 months), and overall survival (OS) (49 versus 148 months) compared to patients without high levels of cPCs [15]. Bianchi et al. then constructed a risk stratification model by including serum M-protein ≥ 3 g/dL as a second risk factor [15]. This study found that patients with no risk factors had a median TTP of 65 months, 30 months for those with one risk factor present, and those with both risk factors present had a median TTP of 12 months [15]. A follow-up to this study using FC instead of a slide-based immunofluorescence assay [16], which is a much more complex and labor-intensive technique, was done with 100 patients diagnosed with the IMWG 2003 criteria from January 2008 until December 2013. Twenty-four (24%) of the patients had cPCs with a median number of 78 cells/150,000 events in those who had cPCs [16]. These patients had a median TTP of 10 months versus not-reached in patients who did not have cPCs [16]. When using a cut-off of ≥150 cPCs to signify a high level of cPCs [16], the median TTP was nine months in the high cPC group compared to not-reached in the low cPC group (<150 cPCs). The positive predictive value (PPV) and specificity of progression at two years were 78% and 97% [16], respectively, for patients with ≥150 cPCs. They also found that the median TTP was 45 months for patients with an i:u FLCr > 8 versus not-reached in the i:u FLCr ≤ 8 group and 60 months in patients with immunoparesis versus not-reached in those who do not [16]. Two multivariate models were developed [16], both contained i:u FLCr > 8 and immunoparesis as risk factors with the presence of cPCs and ≥150 cPCs as the third risk factor for each model, both were found to be independent predictors for two-year progression to MM. When applying Kyle’s 2007 model to Gonsalves’s cohort [10,16], 18 patients were in the high-risk group and had a median TTP of 60 months with the remainder in the intermediate-risk group where the TTP was not reached. The high-risk group in Kyle’s 2007 model had a PPV of only 33% with a specificity of 85% in predicting risk of progression at two years [10]; therefore, the model seems to be not as strong as ≥150 cPCs for predicting risk of progression at two years [10,16].

A study of 96 SMM patients also diagnosed with the IMWG 2003 criteria found a very high-risk group of patients (12.5% of their cohort) who progressed ≤ 18 months from initial diagnosis [17]. Kastritis et al. (2013) sought the risk factors that corresponded to this group of patients and found that a BM infiltration ≥ 60% and a i:u FLCr ≥ 100 had, respectively, a specificity of 95.5% and 98% for identifying patients who progressed ≤18 months [17]. The median TTP for patients with neither of the risk factors was 73 months (10% progressed at 18 months) compared to 18 (66% progressed) and 8 months (100% progressed) for patients with one and both of the risk factors, respectively [17]. A study involving 135 SMM patients diagnosed by the IMWG 2003 criteria showed that all patients with BMPC ≥ 60% progressed to MM within two years [18], but only 64% of patients with a i:u FLCr ≥ 100 progressed. Waxman et al. also developed a risk stratification model with three risk factors [18]: BMPC ≥ 40%, i:u FLCr ≥ 50 and albumin concentration ≤3.5. Due to there being a strong collinearity between FLCr and M-protein concentration [18], the latter was excluded in the model. This is the first model to show that serum albumin levels can be used as a biomarker for progression [18]. The authors stated that this is because levels of serum albumin are inversely proportional to levels of interleukin-6 (IL-6) which is a known growth factor in MM. Patients with zero, one, and two or three risk factors were classified as low, intermediate, and high-risk, respectively [18]. The two-year rates of progression were respectively 16%, 44%, and 81% for the low, intermediate, and high-risk groups, which means the high-risk group and ultra-high-risk group should be offered treatment if the model is validated [18]. Waxman et al. also validated Dispenzieri ’s Mayo Clinic 2008 model and showed that the two-year rates of progression in their cohort was 17, 29, and 69% for, respectively, the low, intermediate, and high-risk groups using the Mayo Clinic model [11], results which are similar to their model except they do not identify an ultra-high-risk group [18]. Wu’s study of 273 SMM patients diagnosed using the IMWG 2003 criteria found that, in their cohort, an i:u FLCr ≥ 100 had a specificity of 90% and a sensitivity of 28% for predicting the progression to MM at two years [19]. In the same time frame, BMPC ≥ 60% had a specificity of 94% and a sensitivity of 15% [19]. However, the median TTP and two-year risk of progression were, respectively, 40 months and 44% for i:u FLCr ≥ 100 [19], while the same measurements were 31 months and 41% for BMPC ≥ 60%.

Gonzalez de la Calle’s study of 147 patients from between 1983–2013 by the IMWG 2003 criteria found that SMM patients with BJ proteinuria had a significantly shorter TTP than those without at 21.7 months versus 82.9 months, respectively [20]. They then were able to divide patients into four different categories with different TTP [20]: 0 mg/24 h (83 months), 1–250 mg/24 h (37 months), 251–500 mg/24 h (16 months), and > 500 mg/24 h (7 months). Using the Danish MM Registry (DMMR), where all newly diagnosed cases of MM (including SMM) in Denmark since 2005 are registered, Sørrig et al. studied 321 patients from 1 January 2005–31 December 2013, who met the IMWG 2010 consensus report criteria and who had no progression/events (including death) in the first three months after diagnosis [21]. They created a risk model using M-protein ≥ 3 g/dL and presence of immunoparesis where the presence of one risk factor was given a score of 1 [21]. The two-year risk of progression was 5, 18, and 38% for the low (score of 0), intermediate (score of 1), and high-risk (score of 2) groups, respectively [21]. Of note, a high FLCr was found to not be a significant risk factor in this study [21].

As mentioned in Section 1, Lakshman et al. sought to develop an improved risk stratification model after the updated IMWG 2014 diagnostic criteria were published [7]. This study included 421 SMM patients seen at the Mayo Clinic between 2003 and 2015 who fulfilled the IMWG 2014 criteria [7]. Lakshman et al. considered several potential risk factors [7]: gender, BMPC% (with a three-year optimal cut-off of 20%), M-protein concentration (three-year optimal cut-off of 2.1 g/dL), i:u FLCr (three-year optimal cut-off of 18.8), M-protein isotype (IgG versus non-IgG and IgA versus non-IgA), and presence of immunoparesis. Lakshman et al. performed a univariate analysis with these factors [7], using BMPC > 20% versus ≤ 20%, M-protein > 2 g/dL versus ≤ 2 g/dL, and i:u FLCr > 20 versus ≤ 20 for simplification, and found that BMPC%, M-protein concentration, i:u FLCr, and the presence of immunoparesis were associated with a shorter TTP. These factors were included in the multivariable analysis where only BMPC%, M-protein, and i:u FLCr were associated with a shorter TTP [7]. They then constructed a risk stratification model, the 2/20/20 model [7], using these three variables. They separated the patients into three groups: the low-risk group with none of the risk factors present, the intermediate-risk group with one risk factor present and the high-risk group with two or three risk factors present, because there was no significant difference in TTP for patients with two or three risk factors [7]. The estimated median TTP was 109.8 months, 67.8 months, and 29.2 months for the low, intermediate, and high-risk groups, respectively [7]. The estimated two-year risk of progression for the three groups was respectively 9.7, 26.3, and 47.4%, but the five-year and 10-year risks of progression were respectively 22.5, 46.7, and 81.5% and 52.7, 65.3, and 96.5% [7]. A 5% risk of progression per year was seen in the low-risk group [7]. This time-independent rate of progression is also seen in MGUS patients (as mentioned in Section 2.1.1). In the intermediate-risk group, the risk of progression was 15% per year during the first two years, 7% per year for the next three years, and 4% per year for the next five years (no measurement was performed past 10 years) [7]. For the high-risk group, the rates of progression were 24% per year for the first 2 years, 11% per year for the next 3 years, and 3% per year for the next five years [7]. This model performed consistently better than Dispenzieri’s conventional Mayo Clinic model with different endpoints for progression (two, three, and five years) when using the same cohort of patients [7,11]. Mateos et al. (2020) validated this model with a cohort of 1966 patients diagnosed after January 2004 from 75 participating IMWG centres in 23 countries [8]. In this study [8], more potential risk factors were included: age (per 10 years), hemoglobin (Hb) concentration, creatinine concentration, calcium concentration, albumin concentration, β-2 microglobulin concentration, light-chain type (Kappa versus Lambda), absolute difference Kappa–Lambda (mg/dL) per 100, heavy chain type (IgG versus IgM and IgG versus IgA), immunofixation, and urine M-protein (mg/24 h) per 1000. Serum M-protein concentration, i:u FLCr, and BMPC% were determined to be the most relevant factors for predicting progression to MM through the stepwise model selection and random forest algorithm [8]. The optimal cut-offs for the risk factors were 1.9 g/dL for serum M-protein, 19.3 for i:u FLCr, and 16.4% BMPCs by Youden’s Index in the receiver operating characteristic (ROC) analyses [8]. Once again, for convenience, 2 g/dL, 20, and 20% were selected as cut-offs for the rest of the study [8]. In their cohort of patients who had information for all three risk factors available (*n* = 1363), the two-year risk of progression was 6.2, 17.9, and 44.2% for the low, intermediate, and high-risk groups, respectively [8]. They also developed a scoring tool [8], which assigned a value to specific ranges instead of just a single cut-off point, where the combined risk score stratified patients into four risk groups (this is elaborated on further in Section 2.2.2 as cytogenetic information is included in this tool). Bruno Paiva, at the 18th International Myeloma Workshop Conference, proposed the replacement of BMPC percentage in this model with circulating tumor cells (CTCs) with a cut-off of 0.7 cells/μL of blood to enhance this model [22].

Using the updated IMWG 2014 diagnostic criteria, Aljama et al. conducted a retrospective study of 306 patients who had a PC proliferative index (PCPI) measurement by bromodeoxyuridine method (patients from 1 July 1996–May 2012) or FC (May 2012–30 June 2016) within six months after diagnosis [23]. Elevated PCPI was defined as >0.5% [23]. This group had a shorter median TTP and a higher two-year risk of progression than those with low PCPI at 3.0 years and 49% versus 7.1 years and 20%, respectively. Aljama et al. then separated patients by the method, and they found that the elevated PCPI group by the bromodeoxyuridine method had a shorter median TTP (3 years versus 6.8 years) and that the difference was not significant, but trending towards significance, for FC (4.7 years versus not reached for low PCPI group, *p* = 0.8), which could have been due to the low number of patients (*n* = 49) in this subgroup [23]. They also tested Dispenzieri ’s conventional Mayo Clinic model and found that the median TTP for their cohort in the low, intermediate, and high-risk groups are 3.4, 5.3, and 11.7 years, respectively [11,23]. They also made two multivariable models using Dispenzieri’s conventional and Lakshman ’s new Mayo Clinic risk stratification models and found that PCPI was an independent factor in both models [7,11,23].

In 2020, the Czech Myeloma Group (CMG) developed a risk stratification model using only serum parameters as these are noninvasive, easy to obtain, and inexpensive [24]. Another reason for only using serum measurements, even though MRI or FC were successfully used in other models, is that some areas of the world have less access to these techniques [24]. Hàjek et al. also wanted to determine whether identifying an ultra-high-risk group with ≥80% risk of progression at two years was possible with this approach [24]. Hàjek ’s study used 287 patients from the Registry of Monoclonal Gammopathies (RMG) of the CMG from May 2007 to June 2013 diagnosed with the IMWG 2003 criteria as a training group (TG) with 240 of the 248 patients (eight from the first study did not give consent to be included in this study) from Neben ’s study (discussed in Section 2.2.2) at the Heidelberg University Hospital, Germany, were used as a validation group (VG) [24,25]. The risk stratification model was developed with presence of immunoparesis, serum M-protein concentration ≥2.3 g/dL, and serum i:u FLCr > 30 as risk factors [24]. The two-year risk of progression for the TG was 18.5, 20.9, 41.9, and 78.7%, respectively, for patients with zero, one, two, and three of the risk factors present [24]. In the VG, the two-year risk of progression was 5.3, 7.5, 44.8%, and 81.3% respectively for patients with zero, one, two, and three risk factors present [24].

Vasco-Mogorrón et al. found that there was an increase in BMPC% and BMPC proliferation rates, but a decrease in apoptotic rates for MGUS, SMM, and MM patients with high risk of progression [26]. They also calculated the proliferation to apoptosis ratio and found that this measurement was a better estimator for PFS than the other three measurements in the study [26]. A ratio of 1.27, with a sensitivity of 51.1% and specificity of 87.1%, was determined to be the optimal cut-off with the highest prognostic capacity for PFS in SMM patients [26]. This ratio was determined to be an independent prognostic factor for both PFS and OS [26]. The ten-year OS was estimated to be 57.5% vs. 35.3% and the ten-year PFS was 82.5% vs. 64.7% for SMM patients with a proliferation to apoptosis ratio <1.27 versus patients with ≥1.27 [26].

A retrospective study of 184 SMM patients found that baseline soluble B-cell maturation antigen (sBCMA) levels, when the cut-off was set to the median value of the cohort (127 ng/mL), had a significantly longer median PFS and OS, at respectively 4.7 versus 1.9 years and 11.9 versus 7.5 years, for patients below the median (<127 ng/mL) compared to the high sBCMA group (≥127 ng/mL) [27]. This study, by Visram, requires independent validation because it used a greater population of patients who progressed to MM than the general population to allow for a comparison between those who progressed and those that did not, which means that the cut-off used may be less sensitive in the general population [27].

Table 1 gives a summary of the models presented in this section. It is difficult to directly compare all of the models since they were published over a span of almost 20 years, which has led to differences in selection criteria due to the evolution of the SMM diagnostic criteria during this timeframe. Furthermore, the differences in published statistics, such as different time frames for publishing risk of progression, also adds some difficulty when comparing these models. It should be noted that the 2/20/20 model is the current recommended risk stratification model, although Bruno Paiva’s model 2/20/0.7 should be monitored for future use [22]. Note that the same classification is not necessarily equivalent between models (e.g., high-risk in Kyle et al. (2007)’s model [10] is not necessarily equivalent to high risk in Pérez-Persona’s model [12]).

#### 2.1.2. Evolving Clinical Measurements

The current SMM risk stratification guidelines do not consider evolving biomarkers; however, several studies have suggested the incorporation of different evolving biomarkers into SMM risk stratification.

Rosiñol et al. identified two subsets of SMM patients showing different levels of risk to progression for the first time [3]. In this study, the evolving group was characterized by a progressive increase in serum M-protein levels [3]. This group also showed a higher proportion of IgA type [3], and more than 50% of the group showed a previously diagnosed MGUS. The evolving M-protein was defined by an increase of the first two follow-up measurements [3]. Most evolving patients had an M-protein increase of ≥10% during the first six months after diagnosis, and all evolving patients could be identified after the first two follow ups [3]. The nonevolving group showed stable M-protein levels that only increased abruptly at disease progression to MM [3]. Interestingly, the evolving group showed a significantly faster median time to progression (TTP) with 1.3 years in comparison to the nonevolving group with 3.9 years [3]. This might indicate that patients with evolving biomarkers are at higher risk of progression to symptomatic MM than patients with non-evolving biomarkers.

Fernandez de Larrea et al. investigated the pattern of evolving M-protein as a risk predictor again with the new SMM diagnosis criteria devised by the IMWG in 2014 (BMPC% ≥ 60%, or FLCr ≥ 100 or >1 focal lesion is now defined as MM) and a larger cohort of patients [28]. The evolving M-protein (eMp) type was characterized by a 10% increase of M-protein levels within one year after diagnosis when patients had a baseline M-protein concentration of ≥30 g/L [28]. If patients had <30 g/L M-protein but showed a progressive increase of M-protein over three years, they were also deemed as evolving [28]. In contrast to the findings of Rosiñol et al. [3], IgA type frequency in the evolving group was not significantly higher than in the nonevolving group in Fernandez de Larrea’s study [28]. TTP was 1.1 years after recognition of the evolving type and patients from the evolving group had a significantly lower OS after progression with 3.4 years compared to the nonevolving type of 6.1 years [28]. This suggests that the impact of the evolving type continues even after progression to MM. The authors confirmed the predictive value of eMp as a high-risk factor and showed that the evolving pattern was the strongest risk factor for progression in a multivariate analysis [28]. All other values (BMPC infiltration of ≥20%, M-protein of ≥30 g/L, or the presence of immunoparesis) were not considered significant risk predictors after the recognition of the evolving type [28].

Lakshman et al. postulated that the characterisation of eMp might reduce specificity [7]. This is because there was no requirement for a minimum absolute rise in M-protein and patients with a low baseline M-protein would only need a very small increase to be characterized as evolving. Ravi et al. conducted a similar study to Lakshman et al. [7,29], where they investigated the impact of evolving changes of M-protein and hemoglobin on risk prediction. In this study, eMp was characterized by a ≥10% increase within six months and/or a ≥25% increase within the first year of diagnosis [29]. A minimum absolute increase of 5 g/L was added to improve specificity [29]. Furthermore, Ravi et al. looked at the evolving changes in hemoglobin levels in SMM patients [29]. A decrease of ≥0.5 g/dl within 12 months of diagnosis was defined as evolving hemoglobin (eHb) [29]. eMb and eHb were both independent risk predictors of progression alongside BMPC of ≥20% within two years after diagnosis [29]. A risk model using those three factors was constructed and median TTP for patients with 0–3 factors was 12.3, 5.1, 2.0 and 1.0 years respectively [29]. The risk of progression within two years was 81.5% in patients with both, eHb and eMp [29].

In a similar study, Atrash et al. used the changes in M-protein levels as well as changes in hemoglobin levels to measure risk to progression [30]. The characterization of eHb and eMp was the same as in the study done by Ravi et al. [29]. eMp was identified as a risk factor for progression alongside an FLC ratio of ≥8 and BMPC ≥ 20% [30]. However, eHb was not a significant factor for SMM risk assessment [30]. The two-year progression rate for patients with eHb and eMp was only 18.5% in this study [30]. This percentage is much lower than the one reported by Ravi et al. [29]. This could be due to the smaller size of the cohort and the lower number of high-risk SMM patients in this study [30].

In contrast to these findings, Wu et al. showed that eHb was a high-risk factor of progression prediction [19]. In addition to eHb, eMP, and edFLC were also identified to be significant factors of two-year progression prediction [19]. dFLC is the difference between involved and uninvolved FLCs [19]. eMP was defined by a >64% increase, edFLC as a >169% increase, and eHb as a >1.57 g/dl decrease, all within 12 months of SMM diagnosis [19].

In a recent study, Gran et al. identified absolute changes in eMp and eFLCr (evolving FLC ratio) rather than relative changes as factors for progression prediction [31]. eMp was characterized as a ≥5 g/L increase and eFLCr as a ≥4.5 increase from SMM diagnosis up to 6 months prior to MM diagnosis [31]. Gran et al. stated that using relative increases in biomarkers as risk prediction factors could overestimate the impact of the biomarker especially because patients with low levels at diagnosis would only need a small absolute increase to be characterized as evolving [31]. A similar argument was previously made by Lakshman et al. [7].

Table 2 provides a summary of models presented in this section. The current recommended risk stratification model is the 2/20/20 model, and not any of the models presented in this section primarily because models based on baseline measurements are better for determining which patients fit the criteria to be enrolled in clinical trials [7]. The use of evolving biomarkers for risk stratification is challenging as the cut-off points between high and low risk SMM are not clearly defined. As stated above, different groups used different MM diagnosis criteria. Some used the 2003 IMWG version and some the updated version from 2014. If newer studies validated older findings while using the current IMWG MM diagnosis criteria, the evolving changes could be added to the current risk stratification models to identify patients showing an evolving versus a stable disease.

#### 2.1.3. Imaging Approaches

Another important biomarker for the risk assessment of SMM is the presence of focal lesions of the bone. Hillengass, et al. used whole-body MRI to determine the prognostic significance of focal lesions in the risk assessment of SMM [32]. The presence of >1 focal lesion was the cutoff point with the highest prognostic significance in this study [32]. Because of the use of whole-body MRI, focal lesions in areas other than the spine were detected in 20% of the patients as well [32].

Kastritis, et al. (2013) confirmed that an abnormal MRI of the spine was associated with a significant risk of progression with a median TTP of 15 months [17]. Furthermore, the abnormal MRI signals correlated with an abnormal FLC ratio of ≥100 (or ≤1/100) and an extensive BM infiltration of ≥60% [17]. Abnormal MRI was also connected to the development of lytic bone lesions at progression to MM [17]. Kastritis et al. (2013) suggested that patients with an abnormal MRI of the spine could be treated with bisphosphates and monitored for the development of focal lesions [17].

In a later study by Kastritis et al. (2014) [33], the median TTP for patients with more than one focal lesion was 15 months. In comparison, the median TTP for patients with no focal lesions was more than five years [33]. These results confirm the importance of MRI of the spine as a factor for risk assessment in SMM. They estimated that the probability of progression to MM is around 70% within two years from diagnosis [33].

Aside from MRI, positron emission tomography (PET) integrated with computed tomography (PET/CT) using glucose labelled with the positron-emitting radionuclide 18F (18F-FDG PET/CT) was investigated by Zamagni et al. as a new tool to identify high-risk SMM patients [34]. Patients with PET/CT positivity had a significantly lower TTP (1.1 years) in comparison to PET/CT-negative patients (4.5 years) [34].

A new approach was the use of 3-D volumetry-based imaging biomarkers derived from whole-body MRI by Wennmann et al. [35]. The speed of growth (total tumor volume over time), characterized by a cutoff of 114 mm^3^/month, showed the highest sensitivity out of all measured biomarkers [35]. Furthermore, it showed a lower false positive rate than the biomarker ‘>1 focal lesion’ which is currently being used by the IMWG [1].

Table 3 summarizes the models presented in this section. Unfortunately, the studies described above do not consider the updated 2014 MM diagnosis criteria in which >1 focal lesion is one of the defining biomarkers for MM. This would mean that patients diagnosed with SMM in the respective studies would now be diagnosed with active MM. However, the last study mentioned revealed a prospective new biomarker, speed of growth observed via whole-body MRI, which would need further validation with a larger patient cohort.

### 2.2. Genetic-Based Models

#### 2.2.1. DNA/RNA Sequencing Approaches and Gene Expression Profiling (GEP)

To find common genetic mutation or mutational patterns within SMM that could define risk groups, studies using genomic analyses have been conducted. Gene expression profiling (GEP) is a common method to identify high-risk MM patients [36]. It was also used in some studies researching SMM risk assessment.

In a study conducted by López-Corral et al. [36], GEP was used to identify four C/D box snoRNA (SNORD) genes (SNORD25, SNORD27, SNORD30 and SNORD31) that significantly correlated with shorter TTP in SMM patients. These genes express snoRNA, a group of noncoding RNA that are involved in post-transcriptional modification of rRNA [36]. According to Williams and Farzaneh [37], some snoRNAs could be actively involved in cancer development. Unfortunately, this study is limited by its small number of patients [36].

Dhodapkar et al. used the risk-score based on a 70-gene signature (GEP70) [38], previously developed by Shaughnessy et al. [39], to identify all major molecular subtypes of MM within their cohort of MGUS and SMM patients. Using this classification, the SMM group included more patients with a GEP70 high-risk score than MGUS [38]. GEP70 was an independent predictor of the risk of progression (2-year risk of progression of 49.7%) in this study [38]. GEP70 > 0.26, M-protein ≥ 3g/dL and iFLC > 25 mg/dL were used to identify a group of patients with high-risk of progression [38]. These findings show that the MM precursor conditions can already be categorized into the different molecular subgroup of MM and shows that the heterogeneity is already present before progression [38]. Dhodapkar et al. suggest that the genetic features of the different subgroup are not likely the key determinants of progression to symptomatic disease [38].

Later, Khan et al. used a gene signature derived from four genes (RRM2, DTL, TMEM48 and ASPM) with a cut-off at 9.28 to identify a high-risk group of patients [40]. They stated that their GEP4 outperforms the GEP70 [40], with a two-year progression of 85.7% for GEP4 vs. 49.7% for GEP70.

Bolli et al. validated the previous findings by Dhodapkar et al. that SMM shows similar genomic landscape to MM [38,41]. This study used whole-genome-sequencing (WGS), a method which has never previously been used to research SMM [41]. It can reveal a lot more than other sequencing methods, such as copy number alterations (CNAs) or genomic rearrangements [41]. The sequenced SMM samples showed similar cytogenetic, mutational, and DNA rearrangement profiles that may also be found in MM [41]. Gain of 1q, del13q, hyperdiploidy, and *IgH* translocations were the most common features found in SMM [41].

Interestingly, Bolli et al. were able to propose two different models of progression to MM [41]. The “static progression model” showed plasma cells that retained the subclonal architecture during progression to MM [41]. The “spontaneous evolution model” showed changes in the subclonal architecture without selective pressure from treatment [41]. The malignant transformation might have been the result of a subclone acquiring a mutational advantage over the other subclones and over a longer period of time [41]. This group showed a significantly longer TTP than the previous one [41].

By analyzing the mutational patterns in SMM, Bolli et al. additionally hypothesized that activation-induced cytidine deaminase (AID) is involved in the early phases of MM development, whereas apolipoprotein B mRNA editing catalytic polypeptide-like (APOBEC) cytidine deaminases drive progression to symptomatic disease [41]. Originally identified as a family of enzymes that edit mRNA [42], it has been discovered that the functions of APOBEC enzymes are very diverse. Some enzymes within the APOBEC family have been shown to produce a mutational signature in cancers [42].

Bustoros et al. used whole-exome sequencing (WES) and showed, again, that most mutations associated with progression to MM are already present in SMM at diagnosis [43]. Risk factors for progression were MAPK pathway (SNVs in *KRAS* and *NRAS*), DNA repair pathway (deletion in 17q and *TP53*, and *ATM* SNVs) and *MYC* rearrangements (translocations or copy number variations of the locus) [43]. Additionally, APOBEC was associated with progression as patients enriched for APOBEC associated mutations had a significantly shorter TTP [43].

Zavidij et al. used single-cell RNA sequencing to investigate the tumor microenvironment of MGUS, SMM, and MM patients to further understand the disease [44]. Increased populations of natural killer cells, T-cells, and nonclassical monocytes were found within patients as early as in the MGUS stage [44]. This indicates that the immune response is already happening very early on in this disease [44]. Furthermore, it was discovered that CD14+ monocytes show compromised MHC-II levels on the cell surface already in the MGUS stage [44], which lead to T-cell suppression when observed in vitro. At the SMM stage, an increase in regulatory and gamma-delta T-cells and a decrease in CD8+ memory T-cells was observed [44]. In vivo experiments in mice, performed by Kawano et al. [45], showed that memory cells play a vital role in tumor immune response. Additionally, IFN signalling was upregulated in patients already in the SMM stage [45]. IFN type-1 has been implicated in immune suppression and MM progression [45]. Seeing an IFN upregulation at the SMM stage might indicate a SMM patient group at high-risk of progression.

Using whole-genome sequencing, Oben et al. identified two SMM groups with different genomic landscapes as well as differences in the temporal acquisition of mutations associated with MM [46]. The progressive group, associated with a higher malignant potential, was characterized by a higher number of genetic myeloma-defining events including “chromothripsis”, template insertions, mutations in driver genes, aneuploidy, and canonical APOBEC mutational activity [46]. In contrast, the stable group that was associated with an indolent course showed a lower mutational burden [46].

Table 4 summarizes the models presented in this section, and Table 5 shows the evolving and nonevolving groups found using DNA/RNA sequencing. While the genomic landscape of SMM can reveal the subclonal architecture, there are no clear markers for the diagnosis of high-versus low risk SMM patients, which would be needed for a robust stratification model. Furthermore, sequencing methods, especially WGS, are still very expensive techniques, and the downstream analysis is complicated.

However, using these methods reveals the heterogeneity of the tumor cells and is very useful to further understand the complexity of SMM and MM. Furthermore, as sequencing costs are decreasing and new techniques such as long-read sequencing are improving, genomic analysis of SMM could add to future SMM risk stratification.

#### 2.2.2. Cytogenetic Approaches

In a cohort of 351 SMM patients from the Mayo Clinic between January 1991 and June 2010, defined by the IMWG 2003 criteria and where a primary molecular cytogenetic subtype could be determined, Rajkumar et al. (2013) found that 43.9% had trisomies, 36.2% had *IgH* translocations, 4% had both trisomies and *IgH* translocations, 15.1% had no abnormalities detected (either normal or insufficient PCs) and 0.9% had monosomy13/del(13q) without any other abnormality [47]. Of the patients with *IgH* translocations [47], 44.9% were t(11;14), 28.3% t(4;14), 8.7% were *MAF* translocations (t(14;16) or t(14;20)), and 18.1% had a different or unknown translocation partner. Patients with t(4;14) were found to have a significantly higher risk of progression as well as a significantly shorter median TTP (28 months versus 55 months) when compared to t(11;14) [47]. The presence of monosomy13/del(13q) did not significantly affect risk of progression and there was a trend towards a higher risk of progression in the del(17p) group (24 months versus 50 months) [47], but there was not a large enough sample of patients (only six) with this abnormality. The patients were then stratified into four cytogenetically distinct groups based on their risk of progression [47]. The high-risk group consisted of patients with t(4;14), trisomies alone were intermediate-risk, standard-risk patients had one of t(11;14), *MAF* translocations, other or unknown *IgH* translocations, monosomy13/del(13q) without other abnormalities, and patients with both trisomies and *IgH* translocations, and the low-risk group was the patients with normal FISH results or insufficient PCs [47]. The median TTP was respectively 28, 34, and 55 months, and not-reached for the high, intermediate, standard, and low-risk groups [47]. Median OS for these four groups was 105 months, 135 months, 141 months, and 135 months [47]. When the high-risk group was changed to include del(17p) [47], the median TTP was changed to respectively 24 months, 34 months, 55 months, and not-reached. The authors state that this modified model is better than the first and is the one they proposed to be used to stratify SMM patients [47]. Another study from the same year, which included 248 SMM patients diagnosed between November 2003 and September 2012, found that the presence of del(17p13), t(4;14), +1q21, and hyperdiploidy had a significant adverse impact on the median TTP, with del(17p13) having the strongest effect [25]. However, del(13q14) and t(11;14) did not have a significant effect on TTP [25]. Patients were stratified into two groups [25], those with one of the high-risk abnormalities (del(17p13), t(4;14), and +1q21) and the standard-risk group (without del(17p13), t(4;14), and +1q21). The TTP rate at three years was 45% in the high-risk group versus 24% in the standard-risk group [25].

Of the 1363 patients from Mateos’s 2020 study [8], 689 also had cytogenetic information available. The presence of t(4;14), t(14;16), +1q, and del13q/monosomy 13 by FISH were the most relevant abnormalities as determined by the stepwise model selection [8]. These relevant cytogenetic abnormalities were added to the risk stratification model as a fourth risk factor [8], which was then separated into four groups with different risks of progression at two years. The low-risk group had no risk factors present with a 6.0% risk of progression, the low-intermediate-risk group had one risk factor present with a 22.8% risk of progression, the intermediate-risk group had two risk factors present with a 45.5% risk of progression, and the high-risk group had three or four risk factors present with a 63.1% risk of progression [8]. The ranges and associated scores for the scoring tool, as described in Section 2.1.1, are shown in Table 6. A score <4 was the low-risk group with a 3.8% two-year risk of progression, the low-intermediate-, intermediate-, and high-risk groups had respectively a score of 5–8 and a risk of progression of 26.2%, 9–12 and 51.1%, and >12 and 72.5% [8].

In our lab, a MM study from 2010–2014, which included 27 SMM patients from CancerCare Manitoba or the Tartu University Hospital, used fluorescence microscopy to analyse the three-dimensional (3D) telomeric profiles of the patients’ BMPCs [48]. We used the TeloView^®^ software (Telo Genomics Corp., Toronto, ON, Canada) [49], which measures the telomere signal intensity (total and average), number of telomere signals, number of telomere aggregates (clusters of telomeres that are unable to be further resolved at an optical resolution limit of 200 nm), nuclear volume, *a/c* ratio (the cell cycle-dependent spatial distribution of telomeres within the nucleus), and the distribution of telomeres relative to the nuclear periphery [48]. All measurements, except telomeres/nuclear volume, were significantly lower among the SMM patients who remained stable for five years (SMM stable) versus SMM patients who progressed within one to three years from diagnosis (SMM with High Risk to Progression) [48].

Table 7 summarizes the models presented in this section. This section showed that cytogenetic abnormalities in SMM are an independent risk factor for the progression to MM and that not all cytogenetic abnormalities are equivalent, some are associated with a higher risk and others a lower risk. Cytogenetic abnormalities also have been shown to improve baseline clinical measurement-based models such as the 2/20/20 model. However, even though cytogenetics should theoretically be recommended, in practice it is much more difficult as not all hospitals or clinical labs have the means and access to perform cytogenetic analysis on their patients. Our lab also showed that cytogenetics-based measurements other than trisomies and translocations can stratify SMM patients and should be studied further. Once again, note that the same classification is not necessarily equivalent between models (e.g., high-risk in Rajkumar et al. (2013)’s model [47] is not necessarily equivalent to high risk in Neben ’s model [25]).

## 3. Treatment of Smoldering Multiple Myeloma

The standard of care for SMM patients is currently observation and not treatment [1]. However, several clinical trials have been conducted to investigate different treatments for patients with SMM with the goal to extend the TTP or even prevent progression to symptomatic MM.

One phase 2 clinical trial conducted by Lust et al. targeted interleukin 1 (IL-1) with inhibitors [50]. First, an in vitro study was done to determine the effectiveness of the inhibitor in IL-6 expressing MM cells [50]. IL-6 has been shown to be essential for the development of myeloma and interleukin 1 (IL-1) β is a major stimulator of its production [51,52]. The disease could be controlled by IL-1Ra alone in patients with less than 20% BMPCs [50], while patients with over 20% BMPCs required IL-1Ra in combination with low-dose dexamethasone. At the conclusion of this trial, IL-1Ra reduced the IL-6 levels and lowered the number of BMPCs [50], while dexamethasone reduced the IL-1β levels in SMM patients. This led to a chronic disease state in patients with an improved PFS [50].

Mateos et al. (2013) performed a phase 3 trial with high-risk SMM patients [6]. Patients were assigned either treatment with lenalidomide and dexamethasone followed by lenalidomide maintenance or observation [6]. Lenalidomide is an immunomodulatory drug and dexamethasone is a glucocorticoid [6]. The median TTP and 3-year survival rate improved significantly in patients receiving the treatment [6], with 77% PFS in the treatment group versus 30% in the control group. OS increased from 80% in the control group to 94% in the treatment group with 90% of the treated patients reaching partial response (PS) or greater during lenalidomide maintenance [6]. The conclusion of this trial was that the treatment with lenalidomide and dexamethasone followed by a lenalidomide maintenance could be used to treat high-risk SMM patients and extend the TTP, PFS, and OS [6].

Mateos et al. (2016) conducted a long-term follow up of the Mateos ’s 2013 study [6,53]. The results confirmed the extended TTP in patients treated with both drugs in comparison with the observation group [53]. Furthermore, dexamethasone was added to the lenalidomide maintenance period in case of progression during maintenance [53]. Disease control was achieved in two-thirds of patients receiving this combination maintenance whereas 64% of patients receiving only lenalidomide maintenance progressed to MM [53].

Mateos et al. (2019) used a treatment strategy called GEM-CESAR to treat high-risk SMM in a phase 2 trial [54]. The strategy consisted of a combination treatment with carfilzomib, lenalidomide and dexamethasone (KRd) [54]. Afterwards, the patients received high-dose therapy-autologous stem cell transplantation (HDT-ASCT), KRd consolidation and lenalidomide and dexamethasone (Rd) maintenance [54]. 56% of patients that completed the whole treatment regimen achieved MRD negativity and 70% reached CR [54].

In another phase 3 trial for SMM by Witzig et al. in patients with SMM [55], thalidomide plus zoledronic acid was tested in comparison to zoledronic acid alone. Thalidomide is an immunomodulatory drug whereas zoledronic acid is a bisphosphonate that can be used to treat and prevent bone complications such as lytic lesions, which are common in patients with MM [55]. The median TTP of patients receiving both drugs was significantly lower than the TTP of patients receiving only zoledronic acid [55]. Interestingly, the 1-year response rate for patients receiving both drugs was 37%, whereas there was no confirmed response for zoledronic acid alone [55]. The combination treatment with thalidomide and zoledronic acid might be able to prolong the TTP in SMM patients [55]. However, the authors mention that this trial was designed before the availability of lenalidomide, which may be a more attractive preventive drug as it may be safer [55].

Korde et al. investigated the safety and efficacy of treatment with carfilzomib, lenalidomide and dexamethasone followed by a lenalidomide extension on patients with newly diagnosed MM (NDMM) and high-risk SMM [56]. The therapy was well tolerated in both patient groups and patients with high-risk SMM showed deeper responses of at least a near complete response (nCR) rate of 100% compared to NDMM patients with 62% [56]. Interestingly, all SMM patients reached at least a very good partial response (VGPR) and minimal residual disease (MRD) was 95% when measured by FC and 75% when measures by NGS [56]. In conclusion, this treatment might be suitable for treating high-risk SMM patients [56].

A phase 2 trial by Ghobrial et al. aimed to determine the effect of elotuzumab in combination with lenalidomide and dexamethasone in high-risk SMM patients [57]. Elotuzumab is an IgG monoclonal antibody that likely stimulates NK cells and that leads to MM cells being killed through antibody-dependent cellular cytotoxicity [58]. The results of the study showed a clinical benefit rate of 97% and an overall response rate of 71%, which may indicate what this treatment could be a suitable treatment to treat high-risk SMM patients [57].

Mailankody et al. treated patients with carfilzomib, lenalidomide and dexamethasone followed by a lenalidomide extension [59]. This is the same treatment regimen treatment as Korde et al. [56], mentioned in the paragraph above. The cohort was larger, and the median follow up was longer than in the previous study [59]. The response rate was 100%, and 63% of patients reached MRD negativity [59]. Furthermore, the genomic landscape of high-risk SMM was compared to NDMM [59]. High-risk SMM showed a significantly lower frequency of mutations in the *NFKB* pathway genes as well as in significant myeloma genes [59]. The authors suggest that these findings could indicate that high-risk SMM shows a better treatment-response biology than NDMM [59]. This supports the early treatment of high-risk SMM patients instead of observation until progression to MM [59].

A new avenue in the treatment of high-risk SMM was the use of a cancer vaccine, which was studied by Nooka et al. [60]. In this phase 1/2 clinical trial, the effect of PVX-410 multiseptated vaccine with or without lenalidomide on moderate or high-risk SMM patients was investigated [60]. The vaccine includes four synthetic peptides from three MM-associated antigens which stimulate cytotoxic T cells that can evoke a tumor-specific immune response [60]. An immune response was observed in 95% of all patients [60], which was higher in magnitude in patients receiving a combination therapy. All patients that received the PVX-410 vaccine alone reached stable disease (SD) [60]. One patient in the combination group reached partial response, four reached SD, and four reached minimal response [60]. The authors suggest that the modest clinical response rates were due to the short duration of the study (12 months) [60]. The results suggest that the vaccine is safe and immunogenic in SMM patients, but further studies with a longer duration are needed to assess the clinical value of this treatment option [60].

In a phase 2 trial by Landgren et al. another monoclonal antibody was tested as a treatment for moderate to high-risk SMM [61]. This study is the basis for an ongoing phase 3 trial of daratumumab on SMM patients. Daratumumab is an IgG monoclonal antibody that targets CD38 [61], which is highly expressed on MM cells. It is currently being used as a treatment for MM, and the authors hypothesize that it might help extend TTP in SMM patients [61]. Patients were assigned either extended intense, extended intermediate, or short dosing schedules [61]. One coprimary end point of CR > 15% was not met during the study [61]. However, the authors specify that the other coprimary end point, which was met, of progressive disease (PD)/death rate and the ORR indicate that daratumumab has single- agent activity in SMM and should be investigated further [61]. Furthermore, results showed that long term dosing of daratumumab delays progression in SMM [61]. Interestingly, the authors support the use of evolving biomarkers for the prediction of risk in SMM and the identification of patients that could benefit from treatment [61].

Lenalidomide has been investigated previously but not as a single agent drug. Lonial et al. performed a phase 3 trial of lenalidomide single agent versus observation with a large cohort of patients with either intermediate or high-risk SMM [62]. The 1, 2, and 3-year PFS rates in the treatment group were significantly higher than in the observation group [62]. The authors support the establishment of early treatment of high-risk SMM patients [62]. Lonial et al. also clarify that the risk assessment should be done using the Lakshman ’s Mayo Clinic 2018 criteria that was validated by the Mateos et al. (2020) [7,8,62].

Table 8 summarizes the clinical trials presented in this section. Observation of SMM still remains the standard of care up to today even for high-risk patients. Fortunately, the 2014 revised IMWG criteria now categorises a portion of these high-risk patients as having active MM. But why are high-risk SMM patients not treated? The heterogeneity of SMM poses a problem because current risk-stratification models are not completely accurate in identifying these ultra-high-risk patients. Furthermore, the discordance between the Mayo Clinic model and the Spanish model is another problem that must be addressed. Treating patients who are not actually high-risk may do more harm than good, so the correct identification of high-risk patients is essential.

Interestingly, recent trials have suggested the use of lenalidomide in combination with other drugs, such as carfilzomib and dexamethasone, as a possible avenue for treatment of high-risk SMM. One ongoing trial is aiming to confirm this possibility (NCT03673826). Moreover, the emerging of alternative drugs such as vaccines and immunotherapy showed very low toxicity and may be a promising start for the early intervention in high-risk SMM patients. Fortunately, many different avenues are currently being explored in ongoing trials. With the improvement of SMM risk stratification models and the identification of safer and more effective drugs, the standard of care for SMM may change from observation to treatment in the future.

## 4. Discussion

There have been many approaches to the risk stratification of SMM patients which are discussed in this review. This includes clinical measurements of biomarkers at diagnosis, evolving clinical measurements of biomarkers, imaging approaches such as MRI, sequencing (which involves WES, WGS, and gene scores, among others), and cytogenetics.

While great improvements have been made to properly identify patients with a low and a high risk of progression, there is still no perfect model where the patients that are characterised as low risk remain indolent, and the high-risk patients all possess aggressive disease progression. Optimizing these models is important because it can help guide physicians on when and how to follow-up with patients [12,13].

More importantly, clinical trials have shown that treating intermediate and high-risk patient with MM drugs and other novel therapies, such as vaccines, have improved patients TTP, PFS, and OS.

A lot of models that have been developed and even used in practice are based around clinical measurements at diagnosis. This is because they are more universal, as there is little to no access to MRI and sequencing instruments, nor fluorescent microscopes in some areas of the world, but also because baseline measurements are more useful for identifying which patients could be enrolled in clinical trials [7,24].

The majority of these studies are retrospective, which can cause certain problems such as missing data, for example FISH or imaging data, which may have led to the inclusion of patients who should have been considered to have MM based on the IMWG 2014 criteria, or simply lowering the sample size for certain risk factors, nonstandard baseline measurement and follow-up times due to changes in clinical recommendations, and a selection bias towards patients who fit certain criteria [1,7,8,14,19,23,27]. However, one of the alternatives, prospective cohort studies, also have problems, namely that such studies are much more expensive and time consuming [13].

In conclusion, developing a risk stratification model that accurately identifies patients who will progress to active MM within two years has shown to be difficult. This is potentially due to the heterogeneity of the disease. However, doing so is necessary as the patients identified as ultra-high risk (≥80%) have been shown to have improved outcomes with treatment. A model that incorporates a large amount, if not all, of the approaches discussed in this paper could, potentially, be the key to a more precise model and better outcomes for SMM patients.

## Figures and Tables

**Table 1:** Summary of baseline clinical measurement models.

Model	Risk Groups	Level of Risk	Reference
Kyle et al. (2007)	BMPC ≥ 10% M-protein ≥ 3 g/dL	High	[10]
	BMPC ≥ 10% M-protein < 3 g/dL	Intermediate	
	BMPC < 10% M-protein ≥ 3 g/dL	Low	
Dispenzieri et al.	BMPC ≥ 10% M-protein ≥ 3 g/dL *κ*/*λ* FLCr < 0.125 or > 8	High	[11]
	BMPC ≥ 10% or M-protein ≥ 3 g/dL and one other of above	Intermediate	
	BMPC ≥ 10% or M-protein ≥ 3 g/dL	Low	
Pérez-Persona et al.	presence of immunoparesis aPC/BMPC ≥ 95%	High	[12]
	presence of immunoparesis or aPC/BMPC ≥ 95%	Intermediate	
	absence of immunoparesis aPC/BMPC < 95%	Low	
Larsen et al.	i:u FLCr ≥ 100	High	[14]
	i:u FLCr < 100	Low	
Bianchi et al.	cPCs ^1^ M-protein ≥ 3 g/dL	High	[15]
	cPCs ^1^ or M-protein ≥ 3 g/dL	Intermediate	
	absence of cPCs ^2^ M-protein < 3 g/dL	Low	
Kastritis et al. (2013)	BM infiltration ≥ 60% i:u FLCr ≥ 100	High	[17]
	BM infiltration ≥ 60% or i:u FLCr ≥ 100	High-intermediate	
	BM infiltration < 60% i:u FLCr < 100	Low	
Waxman et al.	2 or all of: BMPC ≥ 40% i:u FLCr ≥ 50 albumin concentration ≤ 3.5	High	[18]
	BMPC ≥ 40% and/or i:u FLCr ≥ 50 and/or albumin concentration ≤ 3.5	Intermediate	
	BMPC < 40% i:u FLCr <50 albumin concentration > 3.5	Low	
Gonzalez de la Calle et al.	BJ proteinuria > 500 mg/24 h	High	[20]
	BJ proteinuria 251–500 mg/24 h	High-intermediate	
	BJ proteinuria 1–250 mg/24 h	Low-Intermediate	
	BJ proteinuria = 0 mg/24 h	Low	
Sørrig et al.	presence of immunoparesis M-protein ≥ 3 g/dL	High	[21]
	presence of immunoparesis or M-protein ≥ 3 g/dL	Intermediate	
	absence of immunoparesis M-protein < 3 g/dL	Low	
Lakshman et al.	2 or all of: M-protein > 2 BMPC > 20% i:u FLCr > 20	High	[7]
	M-protein > 2 and/or BMPC > 20% and/or i:u FLCr > 20	Intermediate	
	M-protein ≤ 2 BMPC ≤ 20% i:u FLCr ≤ 20	Low	
Aljama et al.	PCPI > 0.5%	High	[23]
	PCPI ≤ 0.5%	Low	
Hàjek et al.	presence of immunoparesis M-protein ≥ 2.3 g/dL i:u FLCr > 30	High	[24]
	2 of: presence of immunoparesis M-protein ≥ 2.3 g/dL i:u FLCr > 30	Intermediate	
	presence of immunoparesis and/or M-protein ≥ 2.3 g/dL and/or i:u FLCr > 30	Low-intermediate	
	absence of immunoparesis M-protein < 2.3 g/dL i:u FLCr ≤ 30	Low	
Vasco-Mogorrón et al.	proliferation to apoptosis ratio ≥1.27	High	[26]
	proliferation to apoptosis ratio <1.27	Low	
Visram et al.	sBCMA ≥ 127 ng/mL	High	[27]
	sBCMA < 127 ng/mL	Low	

^1^ Absolute number of peripheral blood PCs >5 × 106/L and/or >5% PCs/100 cytoplasmic Ig-positive peripheral blood mononuclear cells. ^2^ absolute number of peripheral blood PCs ≤5 × 106/L and/or ≤5% PCs/100 cytoplasmic Ig-positive peripheral blood mononuclear cells.

**Table 2:** Summary of evolving clinical measurement models.

Model	Characteristics of the Evolving Type	Reference
Rosiñol et al.	Progressive increase in M-protein Higher IgA frequency	[3]
Fernandez de Larrea et al.	10% increase of M-protein within one year with baseline M-protein concentration of ≥30 g/L Or <30 g/L baseline M-protein plus a progressive increase of M-protein over three years	[28]
Ravi et al. and Atrash et al.	≥10% increase in M-protein within six months and/or ≥25% increase within the first year with a minimum absolute increase of 5 g/L Decrease of ≥0.5 g/dl hemoglobin within one year	[28,30]
Wu et al.	>64% increase in M-protein >169% increase in edFLC >1.57 g/dl decrease in hemoglobin all within one year	[19]
Gran et al.	≥5 g/L increase in M-protein ≥4.5 increase in eFLCr both from SMM diagnosis up to 6 months prior to MM diagnosis	[31]

**Table 3:** Summary of imaging-based models.

Model	Criteria for High-Risk Group	Reference
Hillengass et al.	>1 focal lesion on whole-body MRI	[32]
Kastritis et al. (2013)	>1 focal lesion on whole-body MRI Abnormal FLC ratio of ≥100 (or ≤1/100) BM infiltration of ≥60%	[17]
Zamagni et al.	PET/CT positivity	[34]
Wennmann et al.	Speed of tumor growth at cutoff of 114 mm^3^/month	[35]

**Table 4:** Summary of DNA/RNA sequencing and gene-expression-based models.

Model	Criteria for High-Risk	Reference
López-Corral et al.	Mutation in four C/D box snoRNA (SNORD) genes (SNORD25, SNORD27, SNORD30 and SNORD31)	[36]
Dhodapkar et al.	70-gene signature (GEP70) > 0.26 M-protein ≥ 3g/dL iFLC > 25 mg/dL	[38]
Khan et al.	GEP4 with a cut-off at 9.28	[40]
Bustoros et al.	Enrichment for APOBEC associated mutations	[43]

**Table 5:** Summary of evolving and nonevolving models using DNA/RNA sequencing.

Model	Characteristic	Model	Reference
Bolli et al.	Retained the subclonal architecture during progression to MM	static progression model	[41]
	Changes in the subclonal architecture during progression to MM	spontaneous evolution model	
Oben et al.	Higher number of genetic myeloma-defining events including “chromothripsis”, template insertions, mutations in driver genes, aneuploidy, and canonical APOBEC mutational activity	Evolving	[46]
	Lower mutational burden	Stable	

**Table 6:** Summary of Mateos et al.’s 2020 risk score model [8]. The sum of scores for each of the four risk factors gave the total risk score for each patient [8]. Patients were then separated into four risk groups based on their total risk score [8]: low, low-intermediate, intermediate, and high-risk.

Risk Factor	Score ^1^
i:u FLCr	-
0–10	0
10–25	2
25–40	3
>40	5
M-protein concentration (g/dL)	-
0–1.5	0
1.5–3	3
>3	4
BMPC%	-
0–15	0
15–20	2
20–30	3
30–40	5
>40	6
FISH abnormality	2

^1^ The higher the score value, the higher the contribution to risk stratification [8].

**Table 7:** Summary of cytogenetics-based models.

Model	Risk Groups	Level of Risk	Reference
Rajkumar et al. (2013)	t(4;14) or del(17p)	High	[47]
	trisomies ^1^	Intermediate	
	one of: t(11;14) MAF translocations IgH translocations ^2^ monosomy13/del(13q) ^1^ trisomies and IgH translocations	Standard	
	no abnormalities detected ^3^	Low	
Neben et al.	one of: del(17p13) t(4;14) +1q21	High	[25]
	without: del(17p13) t(4;14) +1q21	Standard	
Mateos et al. (2020)	3 or all of: M-protein > 2 BMPC > 20% i:u FLCr > 20 relevant cytogenetic abnormality ^4^	High	[8]
	2 of: M-protein > 2 BMPC > 20% i:u FLCr > 20 relevant cytogenetic abnormality ^4^	Intermediate	
	one of: M-protein > 2 BMPC > 20% i:u FLCr > 20 relevant cytogenetic abnormality ^4^	Low-intermediate	
	M-protein ≤ 2 BMPC ≤ 20% i:u FLCr ≤ 20 absence of relevant cytogenetic abnormality ^4^	Low	
Rangel-Pozzo et al.	Low values of 3D telomeric parameters for telomeric profiles ^5^	High	[48]
	High values of 3D telomeric parameters for telomeric profiles ^5^	Low	

^1^ Without other abnormalities. ^2^ Other or unknown. ^3^ Normal FISH results or insufficient PCs. ^4^ One of t(4;14), t(14;16), +1q, and del13q/monosomy 13 present. ^5^ Telomere signal intensity (total and average), number of telomere signals, number of telomere aggregates, nuclear volume, and *a/c* ratio.

**Table 8:** Summary of selected clinical trials.

Group	Treatment Tested	Reference
Lust et al.	Interleukin 1 (IL-1) with inhibitors	[50]
Mateos et al. (2013)	Lenalidomide and dexamethasone followed by lenalidomide maintenance or observation	[6]
Mateos et al. (2016)	Lenalidomide and dexamethasone followed by lenalidomide and dexamethasone maintenance or observation	[53]
Mateos et al. (2019)	GEM-CESAR: combination treatment with carfilzomib, lenalidomide and dexamethasone (KRd), followed by high-dose therapy-autologous stem cell transplantation (HDT-ASCT) and KRd consolidation. Treatment continued with lenalidomide and dexamethasone maintenance	[54]
Witzig et al.	Thalidomide plus zoledronic acid versus zoledronic acid alone	[55]
Korde et al. and Mailankody et al.	Carfilzomib, lenalidomide and dexamethasone followed by a lenalidomide extension	[56,59]
Ghobrial et al.	Elotuzumab versus lenalidomide and dexamethasone	[57]
Nooka et al.	PVX-410 multiseptated vaccine with or without lenalidomide	[60]
Landgren et al.	Daratumumab with extended intense, extended intermediate, or short dosing schedules	[61]
Lonial et al.	Lenalidomide single agent versus observation	[62]

## Data Availability

Not applicable.
